# Part III. Analecta

**Published:** 1827-07

**Authors:** 


### PRACTICAL MEDICINE;

BEING

### The Spirit of the Medical Journals,

*Foreign and Domestic;*

WITH COMMENTARIES.

### PART III.

### ANALECTA.

"Nihil est aliud magnum quam multa minuta."

#### 1. PERIODICAL APYRECTIC DISEASES.

Professor Fulci, of Catania, in Sicily, has published five cases of novel apyrectic affections, of a periodical nature, of which we shall here give some particulars. They are very curious and interesting.

1. The first was a case of tertian and quartan gonorrhœa, in the person of a young law student, who, after impure connexion, became affected with inflammation of the urethra and puriform discharge, for which our professor ordered leeches, emulsions, tepid baths, and low diet. On the second day, he met his patient, who reported himself quite well; but, on the third day, all the symptoms returned, and lasted 24 hours, when again they ceased. The patient a second time reported himself cured, but, on the second day from that period, the gonorrhœa, ardor urinæ, chordee, and all the original phenomena returned as before, and also at the same hour. After this, the disease assumed the quartan type, in which form it made two attacks, and then disappeared in toto. We perceive that the antiphlogistic plan of Broussais and the New School was almost entirely trusted to; for, although some iodine was administered, we cannot attribute the cure to that remedy.

No doubt the mucous membrane of the urethra, like that of the lungs and digestive organs, is subject to intermittent irritations, or even inflammations; we do not, however, remember to have seen any case of the kind recorded by authors.

2. *Cervico-brachial Neuralgia.* A gentleman of Cantorbi, aged 40 years, of nervous temperament and very studious habits, had been subject, for some years, to a chronic bronchitis, for the relief of which he got into the habit of taking opium to rather an excess. Being subject to considerable perspirations, and having caught some cold, he became suddenly affected with severe pain in the cervico-dorsal region of the spine, extending afterwards along the left side of the body, and then fixing itself in the shoulder, shooting occasionally along the arm, and even to the hand of that side. After an accurate examination of the seat of this pain, it appeared evident to our author, that it sometimes followed the divisions of the internal cutaneous brachial nerve—sometimes the course of the external. The sensations experienced by the patient were very various at different times, but almost always of a very distressing kind, causing him to cry out loudly. During these accessions, the muscles of the member contracted violently and involuntarily, and the sufferings were mitigated by pressure, especially by lying on the affected side. The countenance was anxious, and there was great uneasiness in the epigastrium—no sleep—pulse quick. The integuments of the arm affected were two degrees colder than the rest of the body. The paroxysms of pain came on twice in the 24 hours—the first about 11 o'clock, lasting three hours—the other at 10 o'clock in the evening, ceasing towards morning, and giving the patient an interval of repose till 11 o'clock. Vapour baths were employed, under the idea that the neuralgic irritation was occasioned by suppressed transpiration—and the author was afraid of the sulphate of quinine, as there were symptoms of gastric irritation present. Diluents were therefore prescribed, with nitre and mucilaginous drinks. These means mitigated, in some degree, the symptoms; but did not remove them. On the seventh day of the disease a number of leeches were applied to the upper part of the spine. The immediate consequence was, a reduction of the morning paroxysm entirely, and a mitigation of the evening one. The leechings were several times repeated. In twenty days the patient returned home cured.

3. *Mental Alienation, periodical.* A female, aged 40, of highly sanguineous temperament, and very fond of strong liquors and other excesses, started from her bed, one morning, in a state of mental derangement. The phenomenon was attributed by her friends to excesses in drink, and little attention was given to the subject. On the following day she was quite well. On the third day, the same hallucination recurred. Our author was called to the patient, and after an attentive examination of the symptoms, he determined that the disease was an encephalic irritation, accompanied by active sanguineous congestion in the brain, caused by addiction to wine. He, therefore, ordered ten leeches to the temples—cold lotions to the head—warm bath to the lower extremities. Rigorous diet was prescribed, and diluent drinks. Returning next day, our author found the patient quite well in mind, and occupied with her domestic concerns. The most positive injunctions as to regimen were given and observed; but, notwithstanding these, the malady returned next day, although the phenomena of the hallucination were very different from those previously presented. The bizarreries of the patient we shall not enumerate, as they may be as various as the shapes of the clouds, without any real difference in their nature. In this case, the maniacal paroxysm was so violent that no sanguineous depletion, local or general, could be effected. In 22 hours the agitation subsided, and was succeeded by a state of exhaustion and depression, but with complete restoration of reason. The author now saw that he had to treat an intermittent encephalitis, in the form of periodical mania, but determined to let the third paroxysm pass before he attempted to stop its course. This attack came on at the expected period, but with still a new form of mental hallucination. The ideas of the patient, this time, were all of a devotional and penitential character—and she actually forced her husband to repair with her to the church, in order to confess her manifold sins and transgressions. Our author saw her on her return, and, on examination of the other functions of the body, nothing particular could be detected. Her face was rather flushed, and she complained of pain at the summit of the head. A consultation was called, and it was agreed to prescribe the quinine, with cold applications to the head. Twenty-four grains of the quinine were given within the 24 hours. The next paroxysm was on the usual day, but greatly mitigated in severity. When over, the quinine was again administered, and the malady was now entirely checked, and did not return.

Mental alienation has been observed to take on periodical accessions in all ages; but it is rare to see it assume a regular tertian form. The brain, however, being an organ through which both physical and intellectual functions are manifested, we cannot wonder that its derangements should disturb the latter as well as the former class of functions. In such cases it is by physical, not moral remedies, that we can expect to cure the disease.

4. *Periodical Ascites.* A young woman, 25 years of age, of previous good health, good constitution, and affected with no hereditary disposition to disease, experienced a fright and violent mental agitation, soon after which she perceived her abdomen swell, and her urine to become very scanty. A physician was called in, and after attentive examination, he pronounced the disease to be ascites. Before any medicines could be administered, however, the swelling quickly disappeared after an eruption of the menses. The belly again swelled, and again the urine became deficient. Medicines of the diuretic and evacuant kind were prescribed, but without the least effect. The catamenial discharge, as before, was immediately succeeded by a copious flow of urine, and a subsidence of the ascites. This recurred a third time, in spite of various medicines. Our author was now called in, and discovered some signs of gastro-enteric irritation, if not inflammation, with an unusual exhalation from the peritoneal surface. The remedies were, of course, leeches to the abdomen, low diet, and demulcent drinks. External applications of colchicum, antimony, and squills, were also employed over the abdomen. These means failed, and then various physicians, both of Sicily and Italy, were consulted, and exhausted the Pharmacopœia, without the slightest advantage. The ascites commenced immediately after each menstruation, and continued to increase till the next period, when it entirely subsided. The complaint has now continued six years, with the most obstinate regularity, and no remedy has ever interrupted its course.

The above is certainly one of the most curious instances of periodical ascites which we have ever heard of. Stork asserts that he has seen an intermittent ascites, and there are a few others on record; but none, we think, so well authenticated and unequivocal as this one.

5. *Anomalous Intermittent Neurosis.* An ecclesiastic of Catania, aged 55 years, of good constitution, but of a family disposed to hypochondriasis, had led a very regular life. Twenty years ago he became affected with epilepsy, which lasted four years, and then disappeared. Two months ago, the patient, without any known cause, except some bodily fatigue, was suddenly seized with dyspnœa, which obliged him to stop short and sit down quietly, when it ceased. This was considered a thing of no importance, and was not attended to; but the attacks became frequent and severe, and then our author was consulted. On examining the region of the heart, the pulsations were found deep and obscure—the sterno-cardiac region sounded dull—the pulse was irregular—there was inability to lie on the left side—the cheeks were flushed. Prof. Fulci concluded that the disease was angina pectoris, and prescribed accordingly. The attacks of dyspnœa, however, recurred two or three times a day, for six days, and then they disappeared. In these attacks the pulse sometimes fell to 45 in the minute, with strange sense of dragging along the spine, and weakness of the upper extremities.

The attacks of dyspnœa had not ceased more than 24 hours, when a new train of phenomena was developed. In these new paroxysms, the pain darted from the left side of the chest all round the thorax, to the spot whence it first emanated, accompanied by rapid action of the heart and palpitation. The spinal column was also the seat of violent lancinating pain, and the features became decomposed. There was no dyspnœa, and the attack generally lasted about six minutes, returning three times in the 24 hours. Looking upon the complaint, in its present form, as neuritis, leeches were applied along the vertebral column, and counter-irritation to the lower-extremities, with rigid vegetable diet. In eight days the complaint vanished; but in three days afterwards, he was seized with a burning heat along the lumbar region of the spine, accompanied by convulsive movements of the lower extremities, keeping the whole body in a state of agitation. A sense of coldness spread along the thighs and legs—the pulse was contracted and frequent—and there was some tendency to delirium. These paroxysms lasted six hours, and then went off, leaving the patient exhausted and depressed. The next day, and nearly at the same hour, the paroxysm returned, preceded by a sense of tightness in the epigastrium. The periodicity of the complaint being now ascertained, thirty grains of the sulphate of quinine were given in the succeeding interval. The next attack was less violent, and the complaint did not return—at least in that form. In two or three days it made its re-appearance in the shape of a violent sciatica, the pain running from the hip to the foot. These attacks were also periodical, returning diurnally. Narcotics failed—then leeches were employed, with baths and blisters. These also failed—and the disease ultimately gave way to oil of turpentine exhibited night and morning. This last form of the disease harrassed the patient for 28 days, but he has since continued well.—*Bibliotheque Medicale, March,* 1827.

Diseases of an intermittent type are, we apprehend, becoming more prevalent in the present day than formerly; probably from the nervous system being more susceptible in modern times than in the days of our ancestors. The more accurately we observe phenomena in sickness, the more frequently will we find a regular or irregular periodicity in the march of maladies, especially those of the nervous kind.

---

### 2. CEPHALO-SPINAL FLUID.*

In our last Number, (No. xii.) page 592-3, we gave a brief account of M. Magendie's experiments to determine the quantity, nature, use, &c. of the cephalo-spinal fluid. That distinguished physiologist has recently published several memoirs on this interesting investigation, which, indeed, is yet in its infancy. We shall only be able to notice in this place, the substance of his last memoir, read at the Academy of Sciences on the 12th February, of the present year.

In the preceding memoirs, our author shewed that a fluid, formed chiefly in the spinal canal, had a free communication with the cerebral ventricles, by means of an aperture situated near the calamus scriptorius, and that, *vice versa,* the fluid of the ventricles had free exit into the spinal canal. Finally, M. Magendie averred that the fluid formed between the pia mater and arachnoid, over the hemispheres of the brain, had a communication with the ventricles, and, consequently, with the spinal canal. In the portion of memoir now under review, our author comes to the facts on which the foregoing assertions are founded. To these facts we wish to draw the attention of our readers.

A female was brought into the Hospital Necker last year, who had been

* Journal de Physiologie, Janvier, 1827.

seized, the preceding evening, with apoplexy and hemiplegia. She died the same night. The dissection was made by M. Magendie himself, in the presence of M. Delorme and others. The fluid in the cranium and in the spinal canal was tinged with blood, but without any clots. M. Magendie then prognosticated that there was sanguineous extravasation in the ventricles, and accordingly a fibrinous clot was found in the third ventricle, bathed in a reddish serum. This clot resulted from a slight rupture in the thalamus nervi optici of the right side.

We see, then, that the source of the coloration of the cephalo-spinal fluid was placed in the third ventricle. If there were no flux and reflux in this fluid, it is scarcely possible to conceive how this decoloration could have extended to the very extremity of the spinal canal, in the very short period that elapsed between the attack of apoplexy and the death of the patient. The alternate flux and reflux of the cephalo-spinal fluid, corresponding with the action of respiration, would readily account for the transmission of tinge along the canal.

More recently, M. Magendie observed a fact very analogous to the foregoing. A woman died in a very rapid manner of apoplexy, in the Salpêtrière. In this case, the clot was not entirely confined to the ventricle, but extended to the aqueduct of Sylvius, which was considerably dilated. No part of the clot, however, had entered the spinal canal, yet the cephalo-spinal fluid was deeply tinged down to the very sacrum. The following case has afforded our author direct proof that a morbid and accidental fluid formed in the spinal canal may penetrate into the ventricles by the route already described.

A young and robust gardener, who had been exposed to the vicissitudes of the atmosphere, was brought into the Hôtel Dieu, complaining of great pain between the shoulders and along the spine. He had strong fever, but no affection of the head. The disease was considered as acute rheumatism of the muscles of the back, occasioned by exposure to wet and cold, and some relief was obtained by bleeding and the usual means; but the disease persisted, and took on a more serious character. Motion of the arms and lower extremities became almost impracticable—the urine could not be voided, except by the catheter—and, on the second night, the patient became delirious for some hours, in the midst of which he suddenly expired, while attempting to get out of bed. Such an unexpected event excited the curiosity of the medical officers of the hospital to investigate the cause of death.

On dissection, the cephalo-spinal fluid was observed to be wanting and to have been replaced, throughout the whole extent of the spine, by a yellowish and thick pus, of the consistence of jelly. This substance completely filled the sub-arachnoid space, forming a kind of cylindrical sheath for the spinal marrow down to the sacrum. The arachnoid membrane thus distended was sound; but the subjacent pia mater was injected with red. The fourth, the third, and the two lateral ventricles of the brain were found distended with the same substance, somewhat more fluid than that in the spinal canal. On the most attentive examination of the internal surfaces of these ventricles, they were found quite healthy, and without the least trace of softening, or other mark of previous inflammation—affording a strong proof that the morbid fluid had been but a short time in these cavities, and that it had made its way from the spine, causing the sudden and fatal cerebral symptoms. This may be considered, then, as a new species of apoplexy.

The next proposition which is to be supported by facts, is the communication which is said to exist between the general surface of the brain and the spinal canal. Some experiments on animals induced M. Magendie to think that there was no communication between the cerebral surface and the ventricles; but the following case altered his opinions on that point.

A woman, 75 years of age, was suddenly seized with apoplexy, and also paralysis of the right side. In the course of the day the paralysis became general, and the patient died in 30 hours from the invasion of the disease. M. Magendie prognosticated cerebral hæmorrhage in one of the hemispheres, with extravasation of blood into the ventricles. He commenced the dissection by opening the spine, and he found the cephalo-rachidien liquor highly tinged with blood—from which he regarded it as certain that there was a communication between the seat of hæmorrhage and some of the ventricles. The left hemisphere was first examined, and he there found a large extravasation of blood, which he had no doubt extended to the corresponding ventricle. In this he was deceived. The parietes of the ventricle were completely intact, and there was in it only a small quantity of tinged fluid, similar to that which was seen in the spine. There was, therefore, no direct communication between the focus of the hæmorrhage and the ventricle. In the left ventricle there was a considerable quantity of the same kind of tinged fluid. Here our author was greatly embarrassed. On farther examination, it was found that a portion of the blood extravasated had burst into one of the anfractuosities of the hemisphere, and had there occupied the place of the serous fluid naturally existing in such situation. The extravasation was then easily traced to the inferior surface of the brain—to the superior and inferior face of the cerebellum—and, in fact, onwards to the entrance of the fourth ventricle. He was then able to comprehend how the tinge of blood was given to the fluid in the ventricles, and, ultimately, to the whole of the cephalo-rachidien liquor.

The foregoing facts and arguments will probably be one day of considerable importance in the practice of medicine. It is, M. Magendie thinks, almost certain that the ventricular fluid has its source in a secretion from the pia mater covering the spinal marrow. This is rendered the more probable, when we consider that there is no vascular apparatus in the ventricles likely to produce such an abundant secretion; whereas, the pia mater, cranial and spinal, has an extremely active circulation, and, consequently, is in a condition the most favourable for the production of a copious exhalation. Besides, on opening the ventricles of a living animal, we do not observe the formation of any serous fluid; whereas, on laying bare the pia mater, of the head or spine, the formation of the fluid is seen very distinctly.

"If, then, the ventricular fluid comes from the spinal canal, wholly or in part, in those diseases where fluid is found distending the ventricles, it is evident that our remedial measures should be directed towards the spine, as well as towards the head. This proposition I shall follow up by clinical experience and observation."

In the mean time, M. Magendie relates an interesting fact, which we shall here introduce. A horse was brought into the court-yard of the Ecole de Médecine, in order to be slaughtered for experiments, the animal being affected with a peculiar disease, termed in France IMMOBILITY, which consists principally in the loss of all power of moving backwards—and also considerable want of command over the movements forward. The horse was otherwise young, handsome, and strong. M. Magendie begged M. Breschet to turn the animal over to him, with the view of making some attempt to cure the disease. To this M. Breschet consented. Our author justly conceived that the strange affection in question must be connected with some lesion of the spinal marrow; and, under this idea, he applied four immense and powerful moxas along the spine of the animal. These caustics produced dreadful pain, and the animal became so frantic that it was very difficult to restrain him. The combustion, however, was only rendered the more complete by these exertions of the horse, and four deep and large eschars were the consequence. Two days after the application of the moxas the horse began to evince some power of moving backwards, and, in the course of eight days, he backed freely. After this, M. Magendie took him to his own stables, and put him into harness. The horse is quite well, and, if he has any gratitude, he will draw his master most cheerfully to the end of the chapter.

It appears probable to our author that the immobility of the horse, in this case, was caused by the existence of an undue quantity of cephalo-rachidien fluid making pressure on certain parts of the brain during distention of the ventricles. He had laid before the Academy, long ago, some experiments, shewing that, when certain portions (corpora striata) of the brain were excised, the animal was incapable of making any movement backwards, but was, on the contrary, carried forwards by a kind of irresistible impulse. He thinks the horse, in this case, was in a situation somewhat analogous, from the pressure of fluid in the ventricles, and that the moxas caused absorption of the fluid, and removed the disease. Be this as it may, the fact recorded is not the less worthy of remembrance. M. Magendie avers that he has seen several cases where there were unequivocal symptoms of serous effusion in the ventricles, occurring in the cerebral fevers of children, and where these symptoms quickly disappeared, after large blisters to the spine and between the shoulders. One other thing he has particularly observed, since his attention was excited to this subject, which is this; that, in all those patients who died with symptoms of acute or chronic effusions in the brain, there was a remarkable dilatation of the aqueduct of Sylvius, and, consequently, a free communication between the spinal canal and the cerebral ventricles.

From the foregoing facts, M. Magendie considers himself authorised to conclude—

1mo. That the cerebro-spinal fluid is one of the natural humours of the body—and, from its situation and use, one of the most important of these humours.

2do. That it is indispensable to the free exercise of the cerebral and spinal functions.

3tio. That it protects the parts which it surrounds and touches from external violence.

4to. That it influences the functions of the brain and spinal marrow by the pressure which it exercises on these parts—by its temperature—and by its chemical composition.

5to. That at the bottom of the fourth ventricle, opposite the calamus scriptorius, there exists an opening, affording free communication between the cerebral cavities and the spine.

6to. That the ventricles are always filled with the fluid under consideration—that these cavities are capable of containing two ounces, without any inconvenience to the intellectual faculties—but that, when the quantity materially exceeds this, there is derangement, and generally paralysis of the muscular movements, with more or less considerable diminution of the intelligence.

7timo. That it is extremely probable that there is a flux and reflux of the fluid in question from the spine to the ventricles, and from the ventricles to the spine, corresponding with the movements of the brain that take place in the action of respiration.

8. That a fluid accidentally produced in the spinal canal passes readily into the cavities of the brain, and fills those cavities.

9. That a fluid produced in one ventricle passes readily into the others, and from thence passes quickly into the spinal canal.

10. That an accidental fluid, having its source on the surface of the hemispheres, may pass, in a short space of time, into the spinal canal, and also into the ventricles of the brain.

11. That it is very probable that the fluid naturally existing in the ventricles, and also that found in certain diseases, have their principal source in a secretion of the vascular membrane covering the spinal marrow.

We have now only to add that, on chemical analysis, the cephalo-spinal fluid was found to consist of 98 parts in the hundred of water, the other two parts being composed of osmazome, albumen, chloride of soda, subcarbonate of soda, and a very minute trace of phosphate and carbonate of lime.

The most interesting part of this investigation will be that which relates to the passage of a morbid, or morbidly increased quantity of fluid from the spinal canal to the ventricles of the brain, in certain diseases ; for, if this be ascertained, it will greatly influence our modes of treatment, and probably throw some light on points of pathology now involved in much obscurity.

### 3. ACUTE TETANUS.

[Bartholomew's.]

Another fatal instance of this opprobrium medicinæ has been added to the long black catalogue, by Mr. Earle. Stephen Thomas, aged 20, was received into the above hospital, on the 26th of February, having wounded his foot by a rusty nail six days previously. The spasms about the jaw declared themselves the day preceding his entrance, and when examined, it was found that the abdominal muscles were affected. He was freely purged, without any mitigation of the symptoms. The hydrocyanic acid was then given, and ordered to be increased till it produced decided effects. Thirty ounces of blood were also abstracted, with temporary relief. When the dose of Prussic acid was carried to twenty minims, he became easier for an hour and a half, and he slept a short time. A repetition of the same dose failed to afford any respite. He died in convulsions, in the night of the 27th, the day after he came into the hospital.

*Dissection.* The pia mater was rather vascular, and some red points were seen on slicing the brain, which was otherwise healthy, as was the medulla spinalis. The left pleura was extensively inflamed, and the corresponding lung gorged with blood. The sympathetic nerve, in contact with the pleura, was very vascular. The pleura, lung, and nerve, on the right side were not so much inflamed as on the left. The aorta was filled with fluid blood, and there was some fluid in pericardio. There were some other marks of phlogosis in the chest and abdomen, but nothing on which to ground any pathological conjecture as to the nature of tetanus. When the original wound was examined, the internal plantar nerve, before it divides into the two branches which supply the great toe and adjacent one, was completely torn through, and each extremity of the nerve was bulbous and vascular. The theca binding down the tendons of the great toe was wounded.

Mr. Earle suggests the employment of strychnine, in doses to affect the nervous system. Certainly in this disease and in hydrophobia, it is lawful to employ any new and powerful remedy, capable of making a decided impression on the nervous system. We are convinced the whole of the disease consists, at the beginning of tetanus and hydrophobia, in nervous irritation, and that all the changes of structure and vascular alterations which present themselves in the dead body, are merely *effects* of the orgasm in the nervous apparatus. It is to this last that efficient remedies must be applied.—*Med. and Phys. Journ. April.*

### 4. EXTENSIVE ANEURISM OF THE ABDOMINAL AORTA.*

This case occurred in Dr. Elliotson's practice, at Bartholomew's, and is curious from the great size which the aneurismal sac attained. The patient, J. Davis, æt. 33, was admitted Dec. 31st, for pain in the right hip and thigh which was thought to be rheumatic. On the 2d January, the man drew Dr. Elliotson's attention to a large circumscribed and indistinctly pulsating tumour in the right loin, which he said he had only discovered two days previously. No pulsation in the right iliac or femoral arteries, nor indeed in any part of the right limb ; glands in the groins tumefied ; pulse 120 and feeble. Dr. E. now considered the disease aneurism of the abdominal aorta. On the 11th, the patient expired rather suddenly.

*Dissection.* On examining the aorta an aneurism was found, commencing at the crura of the diaphragm, and passing down on the right side, to below Poupart's ligament. The right kidney was pushed into the right iliac region constituting the tumour observed during the lifetime of the patient, the vena cava was forced far away to the right. On the left side the cyst reached only a little below the kidney, which was thrown forward on the vertebral column. The whole circumference of the aorta, from its commencement to the giving off of the renal arteries, was dilated to about double its natural size, but the aneurismal sac came from its back part, and gradually passed over to the right side, extending, as has been mentioned, to below Poupart's ligament. The sac was filled with coagula, not deposited in layers ; of the state of the coats of the artery the reporter is ignorant. The calibre of the stomach and intestines was much diminished, and the sigmoid flexure of the colon was removed to the opposite side of the abdomen. The aneurism had not burst.

### 5. POISONING BY BELLADONNA.

Two interesting cases of this accident happened in the practice of Messrs. Adams and Smith, surgeons, of Torres, N. B. of which the following are the particulars. At 5 P. M. on the 5th November, Mr. Smith was summoned to the assistance of two boys, (brothers) one of two, and the other of three years of age, who, in company with a third child, had eaten of the berries of the Atropa Belladonna, about one or two o'clock of the same day. The elder boy went to school, as usual, but when called up to his lesson, he could not speak. He laughed immoderately, and kept grasping at imaginary objects. He was, therefore, sent home ; but the laughing continued, and his silence was changed into immoderate and incoherent loquacity, with constant bodily motion. Suspicions of the accident were quickly formed, and Mr. S. was sent for. He found the boy laughing and talking alternately—extremities in violent, and almost constant motion—eyes fixed—pupils dilated and insensible to light. Twenty grains of sulphate of zinc were given at twice, in the course of a few minutes, and the action of vomiting promoted by tickling the fauces with a feather. A great deal of reddish matter, containing pieces of the berries, was thrown up. By this time the younger boy was exhibiting the very same symptoms, and the same medicine was exhibited, but only in half the quantity. He threw off the same materials as the other. Solutions of tartar-emetic were given to both, but with great difficulty, as the jaws were firmly locked. The vomiting was kept up until both the boys had thrown off very much of the reddish stuff. Castor oil was then given to each. Meantime the symptoms of poisoning had increased. The muscular motions were incessant—

* Lancet, Jan. 27, 1827.

respiration loud and croupal—faces swollen and red—incoherence the same. Enemata were repeatedly exhibited, to assist the operation of the oil—and some vinegar and water was given in the intervals. 6 *o'clock.* The elder boy's breathing is loud and stertorous—face turgid and swelled—skin cold —muscular motion less strong—pulse not perceptible in the radials. He was put into a warm bath, and while there, the jugular vein was opened, and five or six ounces of blood abstracted. Considerable relief followed. He was put into a warm blanket. The inordinate muscular movements now came on periodically; and, in the intervals, there was a strong disposition to sleep evinced by both boys; but this was resisted by the attendants, till towards the morning of the 6*th*, when indulgence was permitted. Strong coffee, and occasionally vinegar and water were given. 9 *o'clock in the morning of the* 6*th*. The elder boy has loud croupy cough. The other symptoms are still much the same in both patients. Four grains of calomel were given to each; soon after which the younger boy voided about twenty skins of berries, and in the course of the forenoon, he had several fæculent motions. At 2 P. M. the younger boy was found cold and deadly pale, with scarcely any pulse. He was immediately put into the warm bath, and the chest rubbed with flour of mustard, while assafœtida enemata were thrown up. The animal heat gradually returned—the pulse became perceptible. At six o'clock, the same state of collapse returned, and again the same means were employed, in addition to which, warm punch and chicken-broth were frequently given. When taken from the bath, he was wrapped in warm flannels, and kept by the fire side. At half-past 7, he was much revived, and called for some drink. He is still purged, and the stools are watery. It was not till the third day (7*th* November) that the boys became sensible to surrounding objects. Previously to this, they were quite blind, and candles held close to their eyes produced no effect, nor did they seem at all conscious of the light. They evinced much thirst after this, for some time, and had some slight convulsive twitches; but they gradually recovered.

Would the carbonate of ammonia have been more beneficial than the punch, when the alarming symptoms of collapse came on?

---

### 6. ON ASPHYXIA.*

However insignificant the life of man may appear to the philosopher or the moralist, yet such is the tenacity with which people cling to the good and to the bad things of this world, that the renewal of health, and the resuscitation of life, (when suddenly suspended) will always be held in high demand by every class and rank of society. The best modes of resuscitation, in cases of asphyxia from the inhalation of noxious gases, from strangulation, drowning, &c. have engaged the attention of many of our ablest physicians and experimental physiologists—but some doubts and difficulties still remain on this interesting subject. With the laudable view of contributing his mite to the improvement of medical science, M. Leroy has come forward with some researches on asphyxia, of which researches we shall furnish our readers with a condensed account in this article.

Every one is aware that, when atmospheric air is prevented access to the lungs, the heart sends black blood instead of red to the brain, and that death is the consequence. This death, however, is only apparent, in the first instance, and the vital phenomena may be generally revived by timely

---

* M. Leroy. Magendie's Journal, for January, 1827.

and proper means. What are these means? They are of two sorts—those which tend to recall the animal heat, and to rekindle the irritability of the capillary vessels, as exposure to a warm temperature, frictions, fumigations, &c.—and those which are specially directed to the renewal of the respiratory process. The first class of remedial agents is far inferior to the second, though it should not be neglected, for by this class alone, life has been often recalled, when the vital phenomena had ceased. Some of the means ranged under the first class are of doubtful efficacy.

The fumes and the infusion of tobacco have been thrown up with the view of bringing into action the excitability of the intestine; but it is more than probable that the smoke and infusion of this substance act as sedatives rather than stimulants. M. Leroy prefers a current of galvanism directed from the mouth to the anus, in the direction of the alimentary canal. This measure he has tried, with considerable effect, in cases of strangulated hernia.

The grand object, however, is the re-establishment of the function of respiration. For this purpose the insufflation of air has been used ever since the days of Holy Writ. But is there no danger attendant on inflation of the lungs with air? Munro, who made use of a large pair of bellows, directed as much air to be thrown in by a single operation of the instrument, as would distend the lungs, and this precept has been handed down by his followers. But a contemplation of the delicate structure of the lungs, and of the force necessary to press down the diaphragm and dilate the chest, would suggest caution in the employment of this measure. The experiments made by our author will tend to shew that this caution is not unnecessary.

He opened the trachea of rabbits, so as to admit an elastic tube, about a line in diameter, through which he blew in quickly with his own mouth, a certain quantity of air, (about two thirds of what his own lungs contained.) The thorax of the animal was fully distended, and a gurgling noise was heard in the chest. In twenty seconds there were violent convulsions; strong efforts to breathe; and, in less than a minute, the rabbit was dead. On dissection, immediately after death, the right chambers of the heart were found gorged with black blood—the aorta contained the same—the lungs presented several black spots, apparently from extravasation of blood. The air-cells seemed larger than natural. This experiment was repeated on seven rabbits, and on one dog—always with the same results, excepting that, in some cases, there was a slight sanguineous and frothy appearance in the trachea.

This sudden death of the animals was evidently not caused by the mere introduction of *carbonized air* from the experimenter's lungs; for, when the same quantity was blown in *slowly*, the animal did not appear to suffer any inconvenience. After living two or three days with the tracheal fistula, the animal was then killed by the sudden inflation of the lungs, as abovementioned. Our author endeavoured to restore to life the animals which had been thus killed by the forcible insufflation of air but never once succeeded. It was remarked by an able physiologist, who witnessed some of these experiments, that, possibly the same deleterious effects would not have been produced by insufflation, had the measure been employed on animals in a state of asphyxia, instead of on living animals. Our author placed animals in a condition of asphyxia, and then experimented; but the results were the same.

When a tube of half the diameter of the one above-mentioned, was used, the animal's life was not destroyed, and he only experienced some difficulty of breathing. Our author concludes that, if such effects result from forcible insufflation, when the animal is in perfect health, the effects must be still worse where life is suspended, and consequently where the organic textures have lost a portion of their vital cohesion and powers. Is it not probable, suggests our author, that many people have been *prevented* from recovery, in cases of asphyxia, by the forcible insufflation, as performed by bellows, instead of having the vital spark re-kindled by artificial means? However this may be, the experiments of M. Leroy authorise caution in the resuscitative process commonly employed. Our author has invented an apparatus by which the air is more slowly and gradually introduced into the lungs than by the common bellows; but we cannot convey a proper idea of this apparatus without the plates, which may be seen in Magendie's Journal.

---

### 7. HYDROPHOBIA.

At a sitting of the Royal Academy of Medicine on 15th February, M. Léveillé detailed a case of supposed hydrophobia. A grey-hound was observed to gnaw and devour his coverlet, but he lapped water freely. His mistress offered him a lump of sugar, when he snarled and bit her finger. On the 27th of December the animal died. The lady, æt. 40, of a nervous temperament, thought little of the circumstance, when, at the end of January, she was seized, during a meal, with a sudden spasmodic constriction of the pharynx and jaws, so as entirely to prevent deglutition; there was likewise excessive agitation. A thick saliva ran from her mouth, and at the end of four days she died. No examination permitted. This communication gave rise to several remarks. M. Mark detailed the case of a child, which died of hydrophobia in 20 days after the bite, whilst the dog recovered. We are not quite clear (and such was the opinion of one or two of the members) that this was a case of genuine hydrophobia, but let that pass. We all know that the vulgar experimentum crucis of a dog's being mad or not, is his refusing to lap water. Now this is an error. M. Girard stated, and the observation is borne out by the experience of Professor Coleman in this country, that very few rabid dogs indeed refuse water. He observed, also, that the disposition to bite is not so common as is imagined, but we question if this remark be so correct. Mr. Coleman says that the dog will know his master to the last, and though he may bite him, it is only in a moment of irritation, from being unexpectedly touched or roused, and that he seems instantly to be conscious of his fault. The rabid dog, indeed, is excessively irritable, and it is this irritability which makes him so snappish, and disposed to bite whatever is in his way. Another most characteristic symptom of approaching madness is the loss of the peculiar note by which the keeper of the hounds knows each dog. This is followed by a ravenous craving for food and drink, the animal tearing even the mortar or brick from the wall and devouring it. M. Vicery remarked that this pravation of appetite obtains at the commencement of the rutting season, at which time, wounds inflicted by the enraged creature are very liable to be followed by bad symptoms, as tetanus, or even spurious hydrophobia. The imagination, too, has at times a great effect, as an instance of which, M. Barthelemi cited his own case. He had been bitten by an enraged dog, and, for three days, he had all the horror of water and difficulty of swallowing which characterise hydrophobia.

At a recent meeting of the Westminster Medical Society, this subject was introduced by Mr. Mayo. Two points attracted much discussion; the propriety of excising the cicatrix any time after the bite; and relieving the horrible spasm on the glottis by a free opening into the larynx between the thyroid and cricoid cartilages. In support of the first proposal, we may remark, that in many, if not most, of the cases on record, an irritation or inflammatory action appears to have been set up in the cicatrix, just prior to the development of the constitutional symptoms. From this fact, it seems not improbable that the hydrophobic poison lurks for a certain time in the part, producing no effect on the system; and it follows, of course, that the removal of this part will prevent the disease from shewing itself. Even after the symptoms have come on, it is worth while to operate, for, in such a disease, no experiment, provided it be not highly objectionable in itself, is to to be rejected. We think the propriety of opening the larynx was likewise fully established by Mr. Mayo. By it we relieve (if we do no more) a formidable and agonizing symptom; a symptom, too, which appears to play an important part in the fatal termination. In a case of hydrophobia, which happened lately at Guy's Hospital, the bitten limb was amputated. This is certainly removing the cicatrix very effectually, but, as was remarked by Dr. Burder, the shock of so severe an operation might prove detrimental in itself, when the bare excision of a scar would not.* A circumstance was related by Dr. Johnson, which goes to prove that a specific poison is taken into the system and circulated there. A pregnant sow was bitten, and went mad. It was proposed by Mr. Coleman to cut the animal's throat, and save the litter. This was done, and eleven young ones taken from the abdomen, seven of

---

* Since the above was written, the case has been published in detail, in No. 192 of the Lancet. The patient, a fine stout young man, æt. 17, was brought into Guy's about 12 o'clock at noon, April 19th. He had been bitten in the left hand by a dog seven weeks previously, whilst attempting to rescue some children from the animal. The dog itself escaped, and was not heard of afterwards. Nothing was done to the wounds, which bled freely, and were quite healed in the course of a fortnight. On the 17th he went to the Theatre, but felt some head-ache, languor, and sickness, and, on the morning of the 18th, symptoms of hydrophobia unequivocally developed themselves. On his admission, there was seen, on the back of the left hand, an irregular cicatrix, one portion being much more elevated than the other, and having a shining appearance, as if a fluid were contained in it: none, however, issued on puncturing the part with a lancet. The hydrophobic symptoms, convulsive paroxysms, &c. were well marked, the intellectual functions being, in the intervals, unimpaired. It was determined, in consultation, to remove the limb which had been bitten, which was done at 2 P.M. by Mr. Callaway. The paroxysms, during the operation, were very violent, but the loss of blood, which was considerable, seemed to control them in some degree, for a short time afterwards. Soon, however, a kind of re-action took place, and, at 8 P.M. the unfortunate man expired. Throughout the disease the pupil was dilated. The pustules under the tongue were looked for, but none could be found. On dissection, there was rather more serum than usual found in the ventricles; on the right plexus choroides was a firm tubercle, the size of a split pea, slightly increased vascularity on the right side, in the cineritious part of the medulla oblongata, and on the same side of the medulla spinalis. The lung, on either side, was emphysematous, and the pleura pulmonalis was raised, in patches, into vesicles on its surface. The cellular membrane covering the diaphragm was emphysematous in a high degree; the lungs were loaded with blood, and the bronchi filled with a frothy fluid. The upper part of the pharynx was pale, as was the œsophagus in its whole extent; the mucous membrane of the stomach, near its cardiac orifice, was rather vascular; the trachea, near its bifurcation, very much so. No other morbid appearances were discovered. In this case it will be seen that there was evident irritation of the cicatrix. The reporter's remarks are unworthy of notice.

which were saved. In the course of ten or fifteen days they all became hydrophobic, and died. In a case of rabies, we should be inclined to recommend the application of a great number of leeches to the throat and back of the neck, and the *free* exhibition of antispasmodics. These, with the excision of the cicatrix, and, if necessary, the opening of the larynx, are, in our opinion, the means most calculated, as far as we know, to give the patient a chance, desperate though that chance may be. Of course we are here speaking of a case to which the surgeon is not called until some time after the infliction of the wound, or till the disease has appeared.

We believe there are few, if any, well authenticated facts on record to prove that another animal, (not of the canine species, as the horse, the sheep, the cow, &c.) when bitten by a rabid dog, and affected with hydrophobia, is capable of propagating the disease. It is said, indeed, that an Italian monk was bitten by a goat that had gone hydrophobic from the bite of a dog; but the fact wants better attestation. Mr. Coleman has never known an instance of the kind in this country. He has known several instances of men bitten by horses and other animals that were hydrophobic from the bites of rabid dogs; but no bad effects ever ensued.

In respect to the pathology of this terrible disease, we conceive that the phenomena bear us out in supposing that a morbid poison is introduced into the system—that its malignant influence is directed to the centre of the nervous system, but chiefly to the medulla oblongata—that the first symptoms are those of excessive morbid sensibility and irritability of the various nerves of sense, especially those distributed about the glottis—that in a considerable number of cases inflammation supervenes about the base of the brain, the throat, the stomach, and other parts, though the disease sometimes terminates fatally before these marks of inflammation appear. If this view of the pathology be correct, it is evident that the two chief indications of *cure* (when unfortunately the disease has not been *prevented*) will hinge on powerful sedatives and depletion combined:—the former to tranquillize the excessive nervous irritability—the latter to prevent the inflammatory consequences which generally ensue.

### 8. AMPUTATION, DURING INFLAMMATION AND SPHACELUS.

The propriety of such an operation has had advocates, but still more of opponents. The following case is recorded by Dr. Heustis, in the January number of the New York Medical and Physical Journal.

*Case.* A labourer, given to intoxication, had his fore-arm severely jammed, by which accident the muscles upon the inside of the arm were separated from the bones, being mangled and lacerated. In three days, the arm was much inflamed and swollen, while an offensive sanies was discharged from the sloughy surface of the wound. Gangrenous vesicles were rising upon the integuments. An operation, though considered very hazardous, was proposed; but the physician in ordinary delayed it till the next day, when the danger, of course, was greatly augmented. Incipient mortification had extended nearly to the shoulder-joint. It was in the month of August, and in the climate of Albania. The arm was taken off very high up. Upon removing the dressings, on the third day, it was found that mortification had attacked the stump. The sphacelated portions, however, were thrown off, in a few weeks, and the man recovered.

Dr. Heustis considers that such an operation, under such circumstances, might be more dangerous in the lower, and consequently larger members.

### 9. SCIRRHOUS TESTICLE IN THE GROIN.

E. W. æt. 20, of a good constitution, had from birth but one testicle in the scrotum. This was the right, and in the left inguinal region was a tumour, not painful on pressure. On making any violent exertion, however, it increased in size, and pain was felt in the direction of the cord. At the age of five years, W. wore a bandage for a twelvemonth, by the advice of a medical man, who considered the tumour to be hernia. This treatment caused much suffering, and it was discontinued by another surgeon, who pronounced the swelling to be occasioned by the testicle that had not yet descended. At the latter end of June, 1825, after an exertion, he felt a sudden pain in this testicle, which soon became hard and swollen. On his admission into hospital, Dec. 20th, 1825, the left testicle lay in the inguinal canal, was very hard, and formed an oval tumour, in length six inches and nine lines, in breadth six inches and two lines. The tumour was not very moveable, and the testicle was adherent to its coats. From its upper and outer part a kind of cord proceeded along the inguinal canal. The pain, which was incessant, was situated almost entirely at the lower part, and was aggravated by turning on the back. Either sitting or lying on the left side with the thighs bent on the pelvis gave most relief. As the disease was to all appearances scirrhus, extirpation was proposed and performed on the 23d December. The testicle was found to have issued from the inguinal canal, and just at its junction with the cord, it was tightly embraced by the external ring. The canal was slit up, and the cord found to be much hardened and diseased as far as the internal ring. Here it was at least an inch in thickness, and a ligature being placed upon it, the cord was divided below. In performing this operation care was taken to avoid the epigastric artery, but the peritoneum was wounded, and a portion of omentum protruded. The testicle being then removed, the wound was dressed with lint and adhesive plaster. In half an hour hæmorrhage took place, so that it was necessary to open the dressings and tie the bleeding vessel. In the evening inflammatory symptoms arose, and thirty ounces of blood were taken from the arm. Next morning it was necessary to take away twenty-two ounces more. After this all went on well—suppuration was established—on the 15th day the ligature came away from the cord, and on the 30th Jan. 1826, the patient left the hospital. In the course of a month, however, he returned, the cicatrix having given way, and an ichorous discharge flowing from the wound. On examination there was felt a deep hard tumour, and there was great pain in the cicatrix and loins. He would not submit to any treatment, and in a few days left the hospital.

This case of scirrhus affecting the testicle before it had descended is rather curious, but of course there is no reason why the organ should not be so affected, in the groin as well as in the scrotum. It is highly probable that the pressure, by bandages, pads, &c. to which the testicle was submitted, and its exposed situation, predisposed it in a great measure to the disease. The state of the cord precluded any well grounded hopes of success from the operation.

Heidelberger Klinishe Annalen; tome ii. 3e cahier.

---

### 10. SULPHATE OF COPPER IN INFLAMMATION OF THE EXTERNAL TUNICS OF THE EYE.*

Mr. Teale, in an ingenious Essay on "the Tonic Treatment of Inflammation," illustrates some of his positions by a statement of the results

* Mr. Teale. Ed. Journal of Med. Science, No. VI.

which he has observed from the application of the sulphate of copper, in ophthalmia. He had long been in the habit of using this remedy in chronic inflammation of the eye, and was afterwards induced to employ this stimulant in the acute stage, and more violent forms of inflammation of the conjunctiva, sclerotica, and cornea.

"The mode in which it has been employed, consists in lightly sweeping a crystal of that salt three or four times over the everted lower palpebra; the eye-lid is then replaced, and the lachrymal secretion soon distributes the sulphate over the whole globe."

The pain generally subsides in a few minutes; or, if not, warm water gives relief. If this should fail, some warm milk or oil is to be dropped into the eye. In many instances, the vessels may be seen contracting in size, and the interstices between them becoming paler, immediately after the application of the sulphate; but more frequently it is followed by a slight increase of redness, and, in children, the redness is often still farther augmented by violently rubbing the eyes. After a few hours, however, the increased redness subsides, and the vessels become gradually contracted. The original pain of the inflammation abates, the intolerance of light diminishes, and, on the following day, a decided improvement is observable. If the irritation produced by the sulphate be slight, the application may be repeated every second or third day.

Our author has seen this remedy employed, with great benefit, in the purulent ophthalmia of infants and adults, in every stage of the disease. If applied at the commencement of the disease, it frequently arrests its progress—and, in the more advanced stages, it mitigates the violence of the complaint. An advantage of this method consists in the prevention of the granular state of the conjunctiva. The same advantages, says our author, are derived from the application of the sulphate in catarrhal inflammation of the conjunctiva, and in those inflammations which arise from external irritation or violence. Great benefit has also been derived from this application in various morbid states of the cornea, as where it is permeated by red vessels, or affected with chronic or acute ulcers. Where affections of the eye are connected with disorder of the digestive organs, the constitutional affection is, of course, to be attended to, in addition to the local treatment. Neither does the employment of the sulphate preclude the use of other means of a local nature, as leeches, &c.

Mr. Teale makes many interesting remarks on the action of mercury, oil of turpentine, and quinine, in many inflammatory affections, which he explains on the theory that, in inflammation, the capillary vessels are in a state of debility and over-distention. For these remarks we refer our readers to the original paper.

---

### 11. FRACTURE OF A CERVICAL VERTEBRA BY MUSCULAR CONTRACTION.*

This case is detailed in the Archives for March. A soldier, an excellent swimmer, plunged head-foremost into the Sambre. His companions seeing him struggle for some minutes, thought him in jest; but, perceiving that he became motionless, they ran to his assistance and dragged him out. On recovering his senses there was found neither fracture nor dislocation, but the limbs were paralysed—he could not support his head—skin insensible—severe pain in the lower part of the back of the neck, without any external wound—priapism, and frequent desire to make water. The patient

* M. Réveillon, Royal Academy of Medicine, sitting of the 8th February.

stated that, at the moment when he made the plunge he recollected that the water was shallow, and suddenly drew his head back to avoid dashing it against the ground, and that at this instant he lost all consciousness. Delirium came on, and in the night the man died.

*Dissection.* The meninges were of a deep red, and the vessels of the brain itself injected. There was sanguineous effusion around the vertebral column; the spinal canal, *without* the dura mater, which was sound, was full of blood, and, finally, "the body of the fifth cervical vertebra was fractured transversely, a little below its middle, so that the two plates of this bone were separated from the lateral masses."

This case is curious; but it is not improbable that the force with which the person plunged into the water, aided the spasmodic contraction of the muscles in producing the fracture of the vertebra.

